# Ectopic internal carotid artery presenting as an oropharyngeal mass

**DOI:** 10.1186/1746-160X-4-20

**Published:** 2008-08-26

**Authors:** Emmanuel P Prokopakis, Constantinos A Bourolias, Argyro J Bizaki, Spyros K Karampekios, George A Velegrakis, John G Bizakis

**Affiliations:** 1 Department of Otolaryngology, University of Crete School of Medicine, Heraklion, Crete, Greece; 2 Department of Radiology, University of Crete School of Medicine, Heraklion, Crete, Greece

## Abstract

Ectopic internal carotid artery (ICA) is a very rare variation. The major congenital abnormalities of the ICA can be classified as agenesis, aplasia and hypoplasia, and they can be unilateral or bilateral. Anomalies of the neck artery may be vascular neoplasms or ectopic position. Carotid angiograms provide absolute confirmation of an aberrant carotid artery, while EcoColorDoppler (ECD) gives also important information about the evaluation of carotid vassels. Nevertheless Computed Tomography (CT) and Magnetic Resonance Imaging (MRI) of the neck provide spatial information about the adjacent pharyngeal anatomy and are less invasive than angiogram. Injuries to the ICA during simple pharyngeal surgical procedures can be catastrophic due to the risk of massive bleeding. We report a case of a 56 year-old male patient suffering from dysphagia associated with aberrant ICA manifesting itself as a pulsative protruding of the left lateral wall of the oropharynx.

## Background

The congenitally tortuous internal carotid artery (ICA) is an uncommon but important anomaly for the otolaryngologist, to recognize. Numerous descriptions of the anomalies of the greatest vessels of the head and neck, as well as of the ICA have been presented in the literature. The deformities of the ICA have been reported with a large variability of pattern and degree. Some of them determine a dislocation of the ICA that can be found at the level of the pharyngeal wall in some cases. Because of this dislocation, the ICA may cause a widening of the retropharyngeal and lateropharyngeal soft tissues. The ectopic ICA poses a risk during both major oropharyngeal tumor resection and less extensive procedures, such as tonsillectomy, adenoidectomy, and uvulopalatopharyngoplasty. We report a case of a 56 year-old male patient suffering from dysphagia associated with aberrant ICA manifesting itself as a pulsative protruding of the left lateral wall of the oropharynx.

## Case presentation

A 56 year-old male patient was admitted to our service with dysphagia, and malaise that had progressed over the last week. Oral examination revealed an edema at the gingival and the soft palate area, as well as a redness and pulsative protruding of the left lateral wall of the oropharynx. The rest clinical evaluations, as well as the blood tests were normal. Because of the palatal edema, he was administered methylprednisolone per os. No other medication was given.

**Figure 1:**
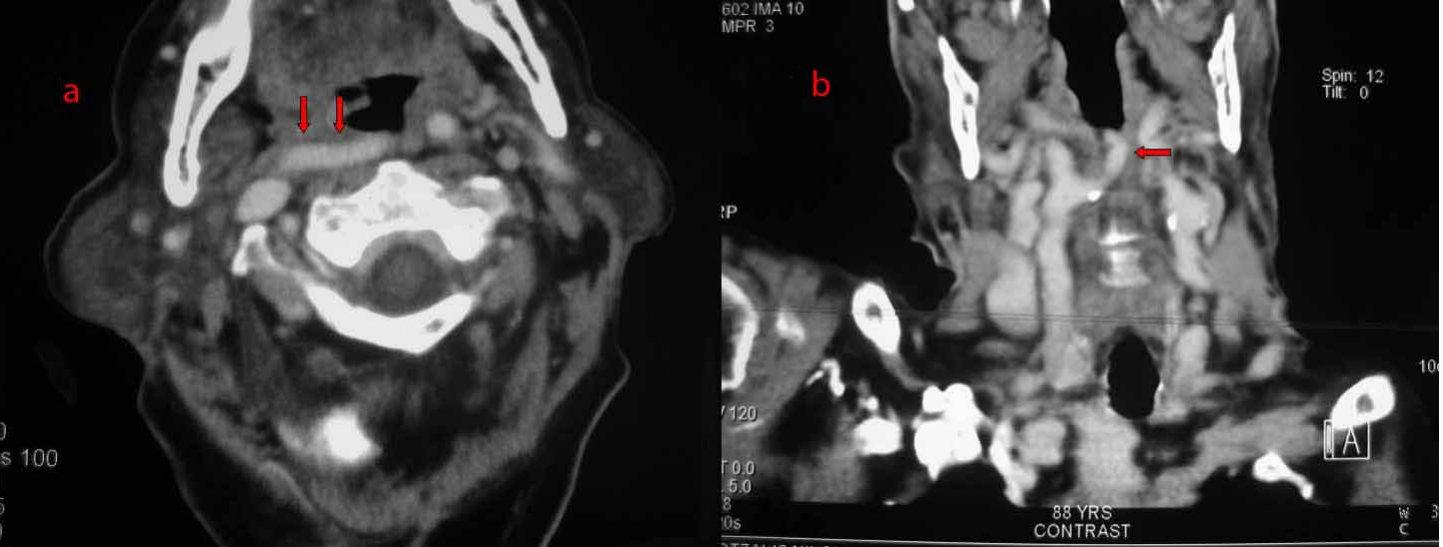
**a. CT scan of the neck, following contrast administration.** Axial section of the level of the oropharynx, demonstrates the horizontal extension of the right ICA towards the midline and behind the oropharynx. b. Multiplanar reconstruction at the coronal plane demonstrates an angiographic appearance of the vessels of the neck, showing the ectopic portion of the right ICA.

A Computed Tomography (CT) of the neck was then performed, which revealed the helicoids, ectopic course of the right internal carotid artery (ICA) at the level of the oropharynx (figure 1a). Multiplanar reconstruction at the coronal plane demonstrates an angiographic appearance of the vessels of the neck, showing the ectopic portion of the right ICA (figure 1b).

**Figure 2:**
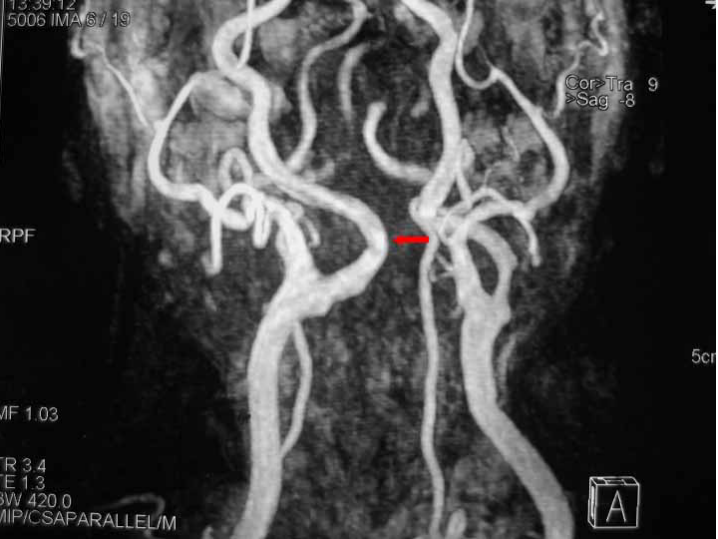
**Magnetic Resolution Angiography after gadolinium administration shows the helicoids-ectopic course of the right ICA, immediately after the carotid bulb.** Notice also, the significant stenosis of the controlateral left ICA.

The abnormal extension of the ICA subsequently was confirmed by Magnetic Resolution Angiography (MRA) of the neck (figure 2). This abnormal course of the ICA was responsible for the gross appearance at the posterior wall of the oropharynx.

## Conclusion

Ectopic internal artery is a very rare variation. The venous anomalies are relatively more frequent than arterials [1]. The ICA originates from the third aortic arch, and it remains controversial whether the common and external carotids have the same third aortic arch origin or they originate from the aortic sac [2-5]. The ICA irrigates most of the cerebral hemispheres and the orbits, and contributes with ramifications to the frontonasal area.

The ICA ascends within the carotid sheath towards the scull base. It is first crossed laterally by the hypoglossal nerve as this nerve passes forward from its position behind the internal carotid. ICA then crosses the occipital artery, as this artery passes posteriorly from its origination of the external carotid artery. Near the skull base the ICA crosses laterally towards the posterior belly of the digastric muscle and the muscle attached to the styloid process. Laterally to the carotid canal is the deep lobe of the parotid gland. Medially to the carotid are the retropharyngeal space and the superior constrictor muscle.

Other vital structures located close to the ICA, are the internal jugular vein, the cranial nerves IX to XII, and the external carotid artery. Inferiorly the internal jugular vein lies laterally to the ICA. The glossopharyngeal nerve passes forward between the internal and external carotid artery at the bifurcation. The hypoglossal nerve passes forward laterally to the internal carotid artery just above the bifurcation. The external carotid artery travels anterior to the ICA throughout its entire course.

The major congenital abnormalities of the ICA can be classified as agenesis, aplasia and hypoplasia, and they can be unilateral or bilateral. Absence of the ICA is referred to as agenesis or aplasia [6].

Anomalies of ICA in the neck may be vascular neoplasms or ectopic position. Vascular neoplasms are more common in children, but two relatively rare neoplasms that occur in the adults are the angiosarcoma and hemangiopericytoma.

The ectopic carotid artery usually occurs in the temporal bone [1]. Angulations of the ICA is a rare condition, while the variations in the course of the carotid artery are divided into two distinct categories: tortuosity and kinking [7]. Elongation, redundancy, undulation, and a S-shaped curve are classified as tortuosity, while any sharp bend in the vessel is classified as kinking. The causes of this malformation are atherosclerosis as observed in our patient, and congenital deformity. The mean age at diagnosis is 58 years, and the patients are usually asymptomatic.

While the reports of fatal posttonsillectomy hemorrhage and the dissections of Kelly clearly describe the unusual laterally placed of the ICA, midline carotid arteries are even less commonly reported [8]. Kelly noted that only four of his 150 patients had posterior pharyngeal wall pulsation. In addition, there are two reports of cases of profuse postadenoidectomy hemorrhage due to laceration of a midline ICA. Mc Kenzie et al described two fatal cases coarsening ICA injuries during adenoidectomy, one of which resulted in complete arterial ablation [9]. Bergqvist described a visible ICA in the nasopharynx that had not been detected preoperatively but was seen intraoperatively after an adenoidectomy had been performed [10].

Ectopic ICAs should be differentiated from other vascular lesions, such as angiosarcoma and hemangiopericytoma. Peritonsillar abscess, masses as lymphomas, and other tumors must be take under consideration, when a panicula in the oropharynx is detected.

We prefer the use of CT or MRI since they are less invasive than angiogram and provide spatial information about the adjacent pharyngeal anatomy. In MRA the resolution of details is not as precise as in angiograms and imaging artifacts due to turbulent flow or patient movement may be a major limitation. Another one examination for the evaluation of carotid vessels is the EcoColorDoppler (ECD), which is easy to perform, and gives quick and important information that MRI and CT do not provide (velocimetry, haemodynamics) [11].

Transposition of the ICA bulging the posterior pharyngeal wall constitutes a risk factor for impressive intraoperative and postoperative hemorrhage in surgical procedure such as adenoidectomy, tonsillectomy, uvulopalatopharyngoplasty and incision of peritonsillar abscess, which are often performed by young and inexperienced ENT doctors. The surgeon should be careful in performing routine surgical procedures in the area of the upper pharynx, which generally represent the most frequent interventions carried out by inexperienced surgeons as the first steps of their surgical training. The hidden presence of an asymptomatic anomaly of the internal carotid artery may cause impressive and life-threatening hemorrhage. In the literature is reported a massive blood loss during tonsillectomy in a child with congenital vascular malformation of the lips and the oropharynx [12].

In our case the referring physician thought that panicula in the lateral wall of oropharynx was edema. The otolaryngologists surgeons must use caution in evaluating patients with masses in the pharynx and augment a careful and complete head and neck examination with appropriate imaging studies before operating. A thorough ocular and digital exploration of the pharynx for arterial pulsations should never be omitted.
